# Chemical Food

**Published:** 1868-06

**Authors:** 


### " CHEMICAL FOOD."

CHEMICAL food has proved a failure from the days of Liebig to the present time. Such is the statement—not the exact words—to be found somewhere in " PROCEEDINGS OF SOCIETIES," as enunciated by one who holds—or once held—that matter is convertible into spirit.

In one of the physiological papers read at the 1867 meeting of the American Dental Association, the writer says, " I repudiate all inorganic food from the laboratory of the chemist." And further: " The particle of phosphate of lime which is intended to become a portion of bone or tooth, must first have received a certain degree of vitality or life by the wonderful force of the VEGETABLE CELL, *before* it can become a living portion of the ANIMAL CELL."

Sentiments similar to these crop out here and there through the profession. When written, they are usually under-scored; when spoken they are as regularly emphasized. If they are true, they need no emphasis; if not true, under-scoring and vehement enunciation can not make them so.

If " chemical food " has only failed since the days of Liebig, it is certainly not chargeable with *prolonged* failure; and if the speaker would show us a specimen of food that is *not chemical*, we would know better how to avoid the universal failure of our race. As we all, with one consent, feed these bodies of ours solely with chemical food, since the days of Liebig as well as before, the failure must be both extensive and serious. But the real failure here is that the speaker has failed to find out what chemical food means, or what the science of chemistry is; and, worse still, he has failed to realize his failure, and is, consequently, a blind leader of the blind on this subject. The nutritive functions assimilate and appropriate, but can not create. They act on matter; and matter is ever obedient to chemical laws, because these are laws of its nature. Vitality modifies chemical action, but never violates chemical laws. Nutrition is a vital function, in which matter changes form, as obedient to chemical laws as are carbon, oxygen, and calcium, in the formation of marble.

Nor are we quite ready to " repudiate all inorganic chemical food." It is still believed that the use of chalybeate water increases the quantity of iron in the red corpuscles. It is pretty certain that the same result may be reached by the use of any one of several compounds of iron, or even of precipitated iron itself. It is usually conceded that common salt, taken into the stomach, will furnish soda for the bile, etc. And if so, these are all cases of assimilation and appropriation, without the "inorganic chemical" substance having "received a certain degree of vitality or life by the wonderful force of the vegetable cell" previous to such change.

We feel satisfied that deeper research will expand and correct the views of some on these subjects. W.

